# Preparation and Characteristics of High-Performance, Low-Density Metallo–Ceramics Composite

**DOI:** 10.3390/ma16247523

**Published:** 2023-12-06

**Authors:** Vitalijs Abramovskis, Reinis Drunka, Štefan Csáki, František Lukáč, Jakub Veverka, Ksenia Illkova, Pavels Gavrilovs, Andrei Shishkin

**Affiliations:** 1 Laboratory of Ecological Solutions and Sustainable Development of Materials, Faculty of Materials Science and Applied Chemistry, Institute of General Chemical Engineering, Riga Technical University, Pulka 3, K-3, LV-1007 Riga, Latvia; pavels.gavrilovs@rtu.lv; 2 Institute of Materials and Surface Technologies, Riga Technical University, P. Valdena iela 7, LV-1048 Riga, Latvia; reinis.drunka@rtu.lv; 3 Department of Physics, Faculty of Natural Sciences and Informatics, Constantine the Philosopher University in Nitra, Tr. A. Hlinku 1, 949 01 Nitra, Slovakia; scsaki@ukf.sk; 4 Department of Horticultural Machinery, Mendel University in Brno, Valtická 337, 691 44 Lednice, Czech Republic; 5 Institute of Plasma Physics of the Czech Academy of Sciences, U Slovanky 2525/1a, 182 00 Prague, Czech Republic; lukac@ipp.cas.cz (F.L.); veverka@ipp.cas.cz (J.V.); illkova@ipp.cas.cz (K.I.)

**Keywords:** cenospheres, syntactic foam, titanium coating, spark plasma sintering, physical vapor deposition

## Abstract

By applying the physical vapour deposition method, hollow ceramic microspheres were coated with titanium, and subsequently, they were sintered using the spark plasma sintering technique to create a porous ceramic material that is lightweight and devoid of a matrix. The sintering process was carried out at temperatures ranging from 1050 to 1200 °C, with a holding time of 2 min. The samples were subjected to conventional thermal analyses (differential scanning calorimetry, thermogravimetry, dilatometry), oxidation resistance tests, and thermal diffusivity measurements. Phase analysis of the samples was performed using the XRD and the microstructure of the prepared specimens was examined using electron microscopy. The titanium coating on the microspheres increased the compressive strength and density of the resulting ceramic material as the sintering temperature increased. The morphology of the samples was carefully examined, and phase transitions were also identified during the analysis of the samples.

## 1. Introduction

Composite materials have been utilised by humankind in various fields for millennia. The progress of modern composite materials continues to advance rapidly due to the escalating demand for lightweight, heat-resistant materials that retain their mechanical strength [1,2]. In aviation, marine, and automotive applications, reducing structural material weight is crucial to meet the future’s rigorous fuel consumption and emissions regulations. With the continuously increasing technological progress demands, traditional materials are inadequate, necessitating the development of lightweight multifunctional materials, such as syntactic foam [3].

Spurious electromagnetic fields can disrupt wireless communications in power systems used by intelligent networks and devices. Electromagnetic interference (EMI) may arise from other devices in the same frequency bands. If this radiation reaches smart devices or the operator’s receiver with sufficient amplitude, it can impact network health, inhibiting innovative system operation and affecting proper wireless transmission [4,5]. 

Three main types of microwave behaviour are typically observed: transmission, reflection, and absorption. Transparent materials, characterised by low dielectric loss, allow microwaves to pass through with minimal attenuation [6,7]. Opaque materials, in contrast, reflect microwaves without transmission. Absorbing materials exhibit high dielectric loss, leading to the absorption of microwaves, the extent of which depends on the dielectric loss factor [8,9]. This absorption is inversely related to both transmission and reflection, resulting in the conversion of microwave energy into heat [10].

Reflection occurs on conductive surfaces, where microwaves are reflected either from the surface or inner layers of the material. The dielectric constant and dielectric loss factor quantify the capacitive and conductive components of the material’s dielectric response. Traditionally, conductive materials like aluminium have been utilised for radiofrequency shielding. However, these materials create a Faraday cage that reflects higher frequency electromagnetic fields, potentially disrupting wireless device connectivity and increasing human exposure to microwave radiation. In areas where mobile communication devices, such as phones and portable computers, employ conductive shielding materials is unjustified. Conversely, absorber materials can neutralise both low and high-frequency electromagnetic fields [11,12].

Numerous studies have explored the modification of construction materials to enhance EMI properties. Examples include special cement composites [11,13,14], carbon foams [15,16,17], SiC/C foam composites [18] (multi-wall carbon nanotube, graphene oxide) [19,20], polymer composites incorporating nanostructured carbon [19,20], with multi-wall carbon-nano tubes [21,22]. As follows from many reviews of EMI shielding materials, the main parameter of the effective EMI shielding material is the presence of a cavity in the material volume, which plays a crucial role in EMI absorbance [23]. Cavity, also called pores, could be open and closed. Porous material with open porosity (usually more than 60 vol. %) is a foam [24,25,26]. Porous material with close porosity (usually more than 60 vol. %) is a syntactic foam [3,27,28]. The majority of the described above materials designed for EMI shielding: (a) build on the polymer base; (b) contain carbon particles made of carbon; (c) made of Al or Mg alloys. Composites with polymer resin matrices—fibreglass, carbon composites (density is from 1.0 to 2.0 g·cm^−3^) cannot withstand temperatures above 400 °C, while those with Mg-Al-matrix (density is from 2.5 to 3.5 g·cm^−3^) are stable until 700 °C. On the other hand, more durable SiC composites possess high-density of 3.0–3.3 g·cm^−3^ making them unsuitable for lightweight applications.

In this research, we are describing the thermal properties of the proposed new materials, which do not contain polymers, carbon, or Al or Mg components and at the same time, have a bulk density below. We proposed material containing mainly ceramic hollow microspheres—cenospheres (CS) and has a syntactic foam structure.

Syntactic foam is a composite material consisting of a matrix filled with hollow microspheres [29]. The microspheres, which are typically made of glass [30,31], ceramic [32,33], or polymer, are dispersed throughout the matrix material, which can be made from polymers [34,35], metals [36,37,38], or concrete and ceramics [39,40,41,42]. Metal foams have lightweight and high-energy absorption properties. The density of the metal foam strongly affects its mechanical properties and deformation mechanism [36,38]. Ceramic foams exhibit exceptional characteristics, including low weight, superior strain and damage tolerance, and excellent thermal shock resistance [43]. The mechanical properties of polymer syntactic foams can be exceptional compared to their low densities due to their ability to absorb significant energy under compressive loading, which is attributed to the microspheres’ resistance to compression. The thermal properties of syntactic foams are generally dominated by the matrix characteristics. The type of the filler also influences the thermal properties of the syntactic foams. If the matrix remains stable the properties of the filler can be preserved up to 400 °C [44].

CS can be coated with metal using physical vapour deposition (PVD). The PVD coatings offer many advantages, such as enhanced surface properties, improved wear and corrosion resistance, and increased hardness [45]. Special attention has been paid to the coating of microspheres with various materials for properties improvement: to increase porosity and decrease density, preserve compressive strength, in some cases catalytic properties, modification of thermal conductivity or dielectric properties, achieve higher thermal resistance than materials which exhibit similar density. Therefore, coatings prepared from different materials such as copper [46], molybdenum oxide [47], and aluminum [48] were developed.

Cenospheres are ceramic particles that are spherical in shape and possess a hollow center and are obtained as by-products of coal combustion [49]. CS sizes are in the interval 40–500 µm [50], which possess a variety of exceptional properties, such as a low heat transfer coefficient, low specific density, high hardness, lack of electrical conductivity, reduced thermal expansion coefficient [51], and complete chemical inertness. Cenospheres are an excellent option as fillers for polymer ceramic composites, catalyst supports, thermal insulation, and chemically resistant layers [41,42,50,52].

This research is a continuation of the previous research [53,54] and has used the concept of matrix-less syntactic foam approach to prepare the samples. The term matrix-less syntactic foam refers to a type of syntactic foam where the filler material is held together without a resin matrix. In previous research, cenospheres were coated with copper [54]; however, in the present research, the properties of the syntactic foam composites made of cenospheres covered with titanium have been examined. As far as the application of this type of material is considered a lightweight thermal protective material, it is necessary to determine the material properties at elevated temperatures—thermal diffusivity, resistance to oxidation, phase composition change, and morphology. 

## 2. Materials and Methods

Cenospheres with a particle size ranging from 63 to 150 µm were used. The properties of cenospheres used in this work were discovered in our previous work [55]. 

Ti coatings on the CS were produced at Ionics Ltd. (Liers, Belgium) an experimental magnetron sputtering setup with a rotating drum for powder-like materials PVD coating. In one batch 5000 cm^3^ of CS was used for coating. Magnetron sputtering system operating at pressures between 2 × 10^−5^ and 8 × 10^−5^ Torr. The process involved constant agitation of the powder reservoir situated below the target by a vibration source to guarantee even coverage. A planar titanium target of 150 mm diameter and an initial thickness of 18 mm was used. The titanium sputter targets had a purity level of 99.9%, which is significant since impurities in the target material can affect the properties of the resulting coating. Ti deposition is controlled by time-to-time taking probe and measuring (by SEM) the deposited Ti layer until it is from 150 to 350 nm.

**Figure 1:**
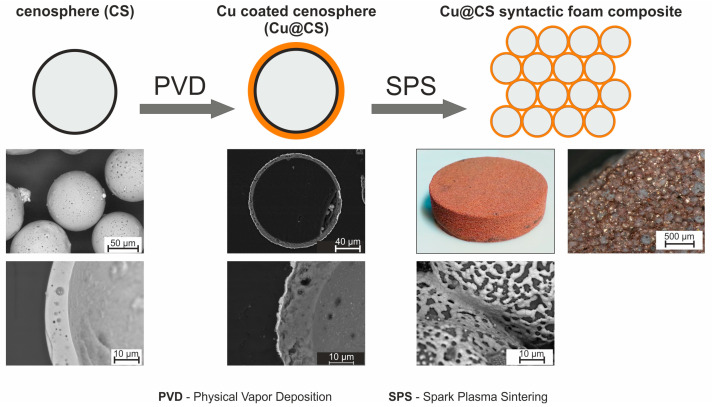
Matrix-less composite sample preparation scheme [54]. Published under Open Access CC BY 4.0 license.

Figure 1 shows a general workflow of the production of the high-performance composite by stages. On the uncoated CS, using the PVD method deposited metal layer was; then, using SPS, it was sintered into single-piece material. Figure 1 gives an example of Cu coating with a thickness of up to 1300 um. In this work, CS coated with Ti layer thickness is from 150 to 350 nm.

The consolidation of the samples for compression tests was accomplished through spark plasma sintering (SPS) using the Toshiba Dr. SINTER SPS-825.CE equipment (Toshiba, Tokyo, Japan). A graphite die having 20 mm and 30 mm in diameter was filled with 7 g (for 20 mm preform) and 14 g (for 30 mm preform) of Ti-coated cenospheres, along with a graphite foil wrapping of 0.6 ± 0.2 mm thickness to prevent the sample from adhering to the die walls. The powder was evacuated and held at a constant pressure of 2.3 MPa for 20 mm samples and 1.7 MPa for 30 mm samples throughout the whole process. The heating rates used were 100 K·min^−1^ for ramp-up and ~200 K·min^−1^ for cooling. The sintering temperature and time were: 1050, 1100, 1150, 1180, and 1200 °C, and 2 min, respectively. Sample core temperatures were acquired pyrometrically through a borehole in the graphite punch. Before mechanical testing and density evaluation, the samples were machined to remove the remaining graphite foil. This caused some variation in sample dimensions and weight, which was accounted for in the determination of both density and compression strength.

The Archimedes method was utilized to determine the bulk density and apparent porosity of the sintered samples. Additionally, compressive strength tests were conducted using the ToniNorm equipment (Toni Technik, Berlin, Germany).

The phase composition of the prepared samples was investigated using X-ray diffraction (XRD) by the D8 Discover (Bruker AXS, Karlsruhe, Germany) diffractometer mounted with the Cu anode and Ni Kβ filter. The angular measurement range was set from 5 to 115° 2θ, with a step size of 0.03° 2θ with 134 s of total time for each step. Phase identification was performed by the X’Pert HighScore software version 4. Quantitative Rietveld refinement was done in TOPAS V5, aiming at the determination of wt.% of all the identified phases following the theory from [56,57].

The sample microstructure was studied using the EVO MA 15 (Carl Zeiss SMT, Jena, Germany) scanning electron microscope (SEM) coupled with energy-dispersive X-ray spectroscopy (EDS) SDD detector XFlash^®^ 5010 (Bruker, Karlsruhe, Germany). Before the SEM observations, the samples were polished using the Struers Tegramin-25 (Struers, Copenhagen, Denmark) with SiC papers up to FEPA P#4000 followed by 1 µm diamond suspension.

Thermal analyses were done to examine the samples’ stability at elevated temperatures. Differential scanning calorimetry (DSC) was performed on the Netzsch DSC404 F3 analyzer (NETZSCH-Gerätebau GmbH, Selb, Germany) in an air atmosphere up to 1100 °C. For thermogravimetry (TG) measurements, the Mettler Toledo TGA/SDTA 851e (Mettler Toledo, Columbus, OH, USA) was used up to 1100 °C in an air atmosphere. Dilatometry (DIL) was performed on the Netzsch DIL 402C analyzer up to 1100 °C in a nitrogen atmosphere. All the thermal analyses were performed at a heating rate of 10 °C/min. 

Oxidation resistance was studied with the help of the Mettler Toledo TGA/SDTA 851e in the air atmosphere. The samples were heated at 10 °C/min to the desired temperature (900, 1000, and 1100 °C) where a dwell time of 5 h was applied. 

The thermal diffusivity of the samples was measured using the Linseis LFA 1000 analyzer (Linseis Messgeräte GmbH, Selb, Germany) in a vacuum up to 1100 °C in 200 °C steps. 

All the analyses were performed at least on 2 samples. Where indicated, a third measurement was done to assure the repeatability of the results. 

To examine the morphology and microstructure of the samples after mechanical tests, scanning electron microscopes from Hitachi (S4800) (Tokyo, Japan) and Mira/Tescan (Brno, Czech Republic) were employed. Additionally, an optical imaging Keyence VHX-2000 digital microscope (Keyence, Osaka, Japan), equipped with VH-Z20R/W and VH-Z500R/W lenses, was utilized.

## 3. Results and Discussion

### 3.1. Microstructure of the Samples

The phase analysis performed using the XRD measurements (Table 1) revealed that the mullite was the main crystalline phase in the samples. With an increasing sintering temperature, the amount of mullite decreased. Nonetheless, an increase in the sintering temperature to 1200 °C led to an increase in the mullite content. The cristobalite started to appear after sintering at 1150 °C and its presence became evident as the sintering temperature reached 1180 °C and remained unchanged when the sintering temperature increased to 1200 °C. Titanium, which was used to coat the individual cenospheres is susceptible to oxidation if oxygen is present at elevated temperatures. Stemming from this, pure Ti was not identified in the samples. However, lower titanium oxides TiO and Ti_2_O_3_ were present at all sintering temperatures. The amount of Ti_2_O_3_ increased as the sintering temperature rose. The Ti oxidation was linked to the presence of air in the sintering chamber (and in sample pores). A moderate amount of amorphous phase was already observed in the sample sintered at 1050 °C. However, the amount of the amorphous phase rose with the sintering temperature up to 1150 °C (50 wt.%), which was followed by a decrease at 1180 °C (43 wt.%) and reaching 38 wt.% at 1200 °C. 

**Figure 2:**
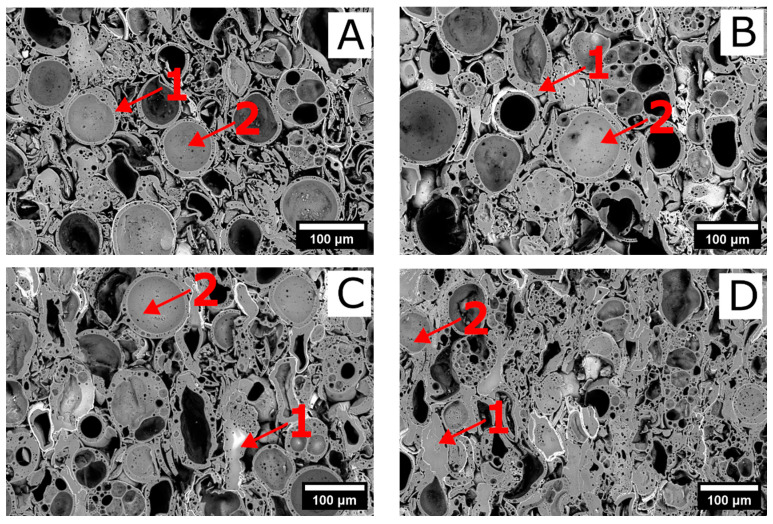
SEM micrographs of the prepared samples ((**A**)—1050 °C, (**B**)—1100 °C, (**C**)—1150 °C, (**D**)—1200 °C). Numbers indicate the EDS sampling places.

The microstructure of the sample sintered at the lowest temperature (1050 °C) was porous, while the Ti-coated cenospheres remained identifiable (Figure 2A). The cenospheres were trapped in an Al-Si matrix. With an increase in the sintering temperature, the pores remained present (closed porosity) and the Ti-coated cenospheres were still identifiable (Figure 2B). Even though according to the XRD results the amount of the amorphous phase did not increase significantly, the differences in the sample’s microstructures were negligible. The Ti coating was still present, however, composed of lower titanium oxides. Nonetheless, as the sintering temperature reached 1150 °C the pores, as well as the cenospheres, became flattened (Figure 2C). Some of the cenospheres retained their spherical geometry. Increasing the sintering temperature to 1180 °C led to a significant change in the microstructure (Figure 2D). A more compact microstructure was formed, however, with closed pores still present in the sample. Both the pores and the cenospheres were flattened. Plastic deformation of cenospheres was also observed in the case of Cu-coated spheres [53].

EDS analysis was performed on the cenosphere surface as well as on their walls (Table 2). The surface of the cenospheres contained a higher amount of Ti in all cases, nonetheless, in the form of oxides. The most abundant element identified by the help of EDS was oxygen in all samples. The presence of Al and Si indicated that the amorphous phase was glassy and formed from aluminosilicates. 

**Figure 3:**
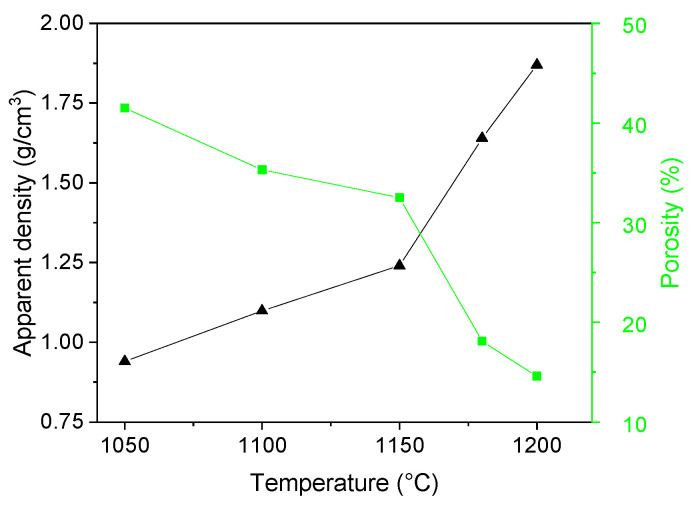
Apparent density and porosity of the samples.

At the lowest sintering temperature of 1050 °C the sample exhibited an apparent density of 0.94 g·cm^−3^ (Figure 3). With an increase in the sintering temperature up to 1150 °C a linear-like increase in the apparent density of the samples was observed. A further increase in the sintering temperature to 1180 and 1200 °C led to a steep increase in the apparent density. At the highest sintering temperature, the sample manifested an apparent density of 1.87 g·cm^−3^, twice as high as at the lowest firing temperature. The aforementioned results were supported also by the SEM micrographs, where a gradual decrease in the porosity was observed. This was ascribed to the formation of a glassy phase, which exhibited lower viscosity at higher temperatures and assisted the densification process. Nonetheless, closed porosity was still observable even after firing at 1180 °C (Figure 2). The open porosity (water accessible)—hereafter referred to as “porosity”—manifested a decreasing character with an increasing sintering temperature (Figure 3). At the sintering temperature of 1050 °C the value of porosity reached 41%. However, as the sintering temperature increased the porosity decreased and at the highest sintering temperatures 1180 and 1200 °C reached values of 18% and 15%, respectively. 

### 3.2. Thermal Analyses

**Figure 4:**
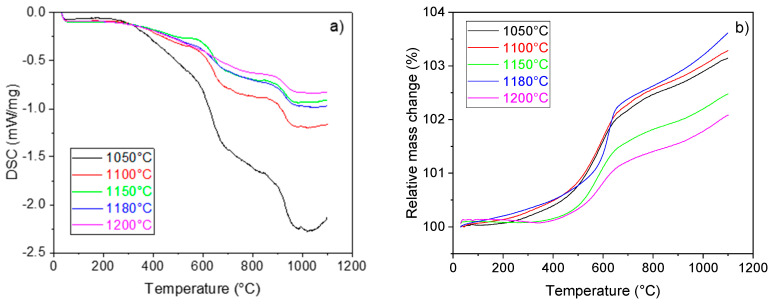
DSC (**a**) and TG (**b**) curves of the samples.

Upon initial heating up to 500 °C no physico-chemical reactions were observed in the samples, according to the DSC curve (Figure 4a). On the contrary, the mass of the samples (Figure 4b) gradually increases in this temperature region. The mass of the samples sintered at 1150 and 1200 °C exhibited an almost unchanged mass up to 400 °C. A steep increase in the sample mass starting at 450 °C represented the start of the oxidation of lower titanium oxides represented by the following formulas [58]

(1) $$TiO+\frac{1}{2}O_{2}=TiO_{2}$$

(2) $$\frac{1}{2}{Ti}_{2}O_{3}+\frac{1}{4}O_{2}=TiO_{2}$$

The presence of the aforementioned lower titanium oxides was confirmed by XRD analysis. After reaching the temperature of ~600 °C, the increase in the sample mass became less steep. Nonetheless, the increase was still evident, as in the presence of oxygen titanium and its lower oxides tend to form TiO_2_. The total increase in the sample mass ranged from 1.8 to 3.6% for the samples sintered at 1200 and 1180 °C, respectively. The dependence of the total mass gain upon heating to 1100 °C on the sintering temperature was not evident. The sintering of the clay cenospheres was observed on the DSC curve to start at ~900 °C. An onset of an exotherm on the DSC curve of the samples sintered at 1050 °C was linked to the possible crystallization processes in the sample.

**Figure 5:**
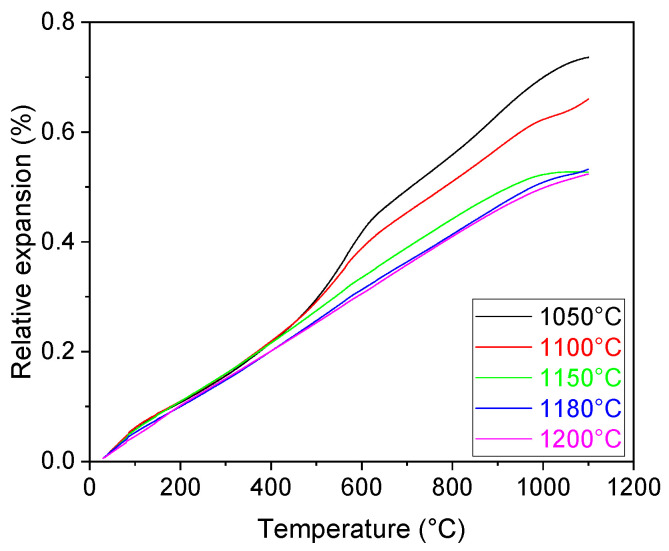
DIL curves of the samples.

A continuous thermal expansion of the samples was observed on the dilatometric curves up to 500 °C for all sintering temperatures (Figure 5). Samples sintered at temperatures 1050 and 1100 °C exhibited a more pronounced expansion in the temperature region from 500 to 700 °C. The overall thermal expansion of the samples reached values ranging from 0.5 to 0.7% up to 1100 °C. Although no evidence of sintering was observed on the DIL curves, it cannot be ruled out. Considering the sample composition, the DIL curves appear to be a superposition of the titanium and its oxides and the cenospheres. It was evident, that once the sintering temperature reached 1150 °C, its effect on the thermal expansion of the samples was negligible. 

### 3.3. Thermal Diffusivity

**Figure 6:**
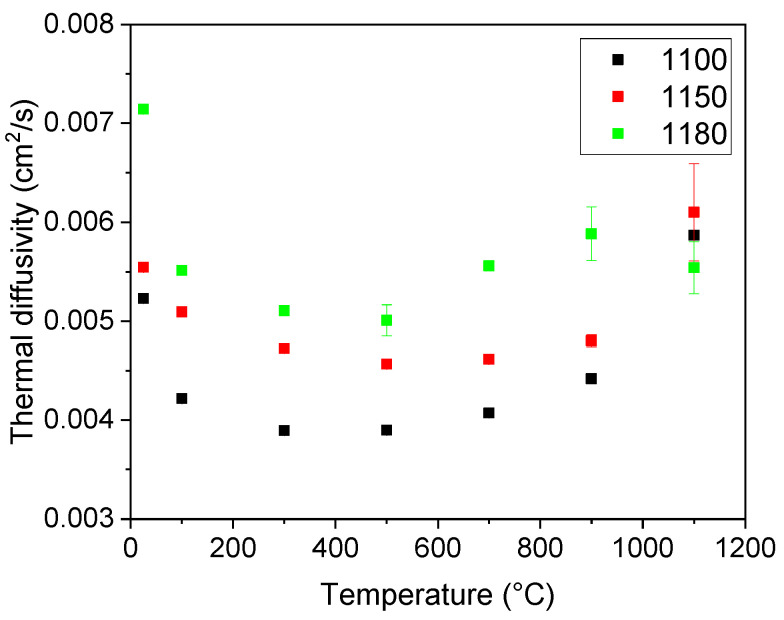
Thermal diffusivity of the selected samples.

For thermal diffusivity measurements, three samples were selected, namely, samples sintered at 1100, 1150, and 1180 °C (Figure 6). At room temperature, a strong dependence of the values of thermal diffusivity on the sintering temperature was observed. This dependence followed the increase in the density with an increasing sintering temperature. The reason for this lies in the porosity of the samples—the more air-filled pores there are present in a sample, the lower the thermal diffusivity, as the solid network exhibits higher thermal diffusivity and conductivity values than air. Stemming from this, the increase in the values of the thermal diffusivity with an increasing sintering temperature (and in turn decreasing porosity) is reasonable. With an increasing temperature, the values of the diffusivity decreased up to 500 °C, where a global minimum for all samples was reached. As the temperature further increased the diffusivity also rose. The differences remained evident up to 900 °C. At the highest temperature, the differences in the values of the thermal diffusivity became negligible. For a complex sample, the final value of the thermal diffusivity is influenced by the individual components. In the present case, the thermal conductivity of the samples is supported by the titanium oxide content. On the other hand, mullite and other ceramic phases exhibit low thermal diffusivity, and therefore their presence hinders overall diffusivity. In addition, the presence of the air and vacuum-filled pores (both open and closed porosity) manifested a considerable barrier for both phonon and electron heat conduction. 

### 3.4. Oxidation Resistance

**Figure 7:**
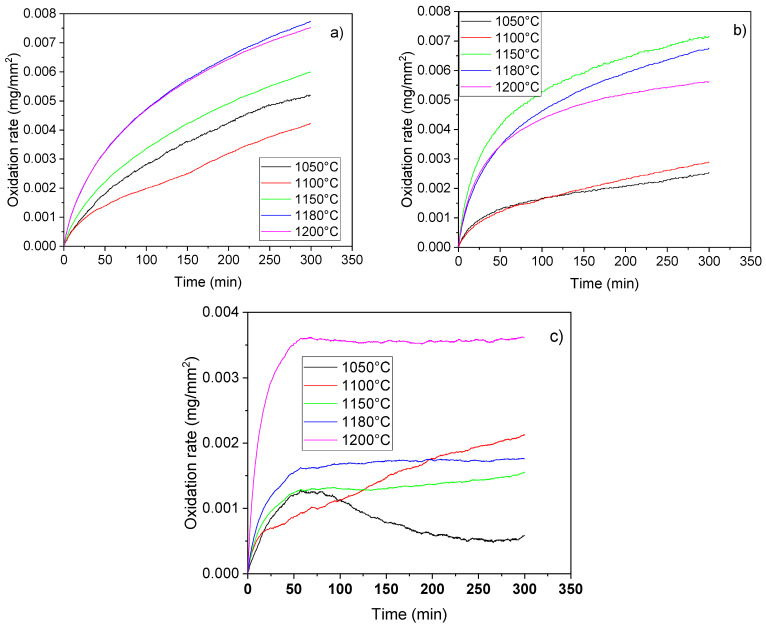
Results of the oxidation tests of the samples at 900 °C (**a**), 1000 °C (**b**), and 1100 °C (**c**).

Samples were exposed to high-temperature oxygen tests for 5 h at three different temperatures, namely 900, 1000, and 1100 °C (Figure 7). At 900 °C the highest oxidation rate was observed for samples sintered at 1180 and 1200 °C. However, TG results showed that the oxidation process had already started at this temperature. The lowest oxidation rate was exhibited by the samples sintered at 1100 and 1050 °C. After 5 h of isothermal heating, the samples’ mass was still not constant, indicating a non-finished oxidation. The observed oxidation was related to the Ti content in the samples. Lower Ti oxides were formed already during the sintering. Nonetheless, these oxides are prone to oxidation, which leads to the formation of TiO_2_, which in turn leads to an increase in the sample mass. However, owing to the low initial Ti content, the oxidation was not intense. Increasing the temperature to 1000 °C led to a change in the oxidation rate for the samples sintered at 1050 and 1100 °C. The aforementioned samples after an initial steep increase manifested a slow gradual increase in the sample mass. Samples sintered at 1150, 1180, and 1200 °C exhibited a parabolic increase in the sample mass. Nonetheless, samples sintered at 1150 and 1200 °C reached an almost constant value after 5 h of heating. The oxidation therefore was intense in the early stages of isothermal heating. A further increase in the temperature led to a complete change in the oxidation rates. All the samples, except for the sample sintered at 1100 °C, reached a constant mass after 50 min of heating. Thus, the stabile TiO_2_ oxide was formed in the sample in a short time at these temperatures. The sample sintered at 1050 °C exhibited a decrease in the sample mass after 50 min of heating, indicating the evaporation of gaseous substances from the sample body. The sample sintered at 1100 °C exhibited a linear-like increase in its mass during the whole studied temperature interval. 

Given the sample composition, only the Ti was prone to oxidation. Although lower Ti oxides were formed already during the sintering, these oxides reacted with the oxygen content of the atmosphere and TiO_2_ was created. Therefore, the oxidation of the samples occurred both during the heating and isothermal stages leading to a slow oxidation rate during the isothermal heating. Once the TiO_2_ is formed no further oxidation is observed, and, therefore, the samples exhibited good thermal resistance. 

### 3.5. Mechanical Properties

**Figure 8:**
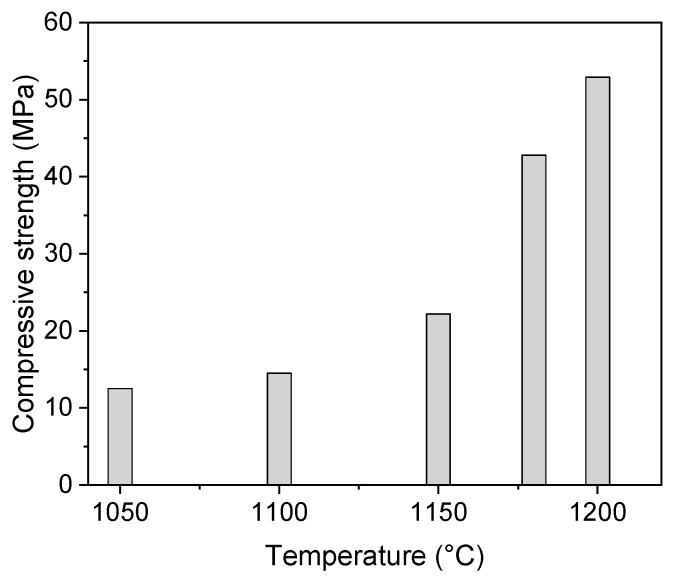
Compressive strength of the prepared samples.

The sintered samples were subjected to a measurement of compression strength (Figure 8). At the lowest sintering temperature, the value of the compressive strength reached 12.5 MPa. Nonetheless, with an increase in the sintering temperature, the sintering of the samples proceeded, and a more compact microstructure was formed (Figure 2 and Figure 3). Consequently, the mechanical properties of the samples improved. Up to the sintering temperature of 1150 °C the increase was only moderate. Nonetheless, the value of the mechanical strength reached 43 and 53 MPa after sintering at 1180 and 1200 °C, respectively. 

**Figure 9:**
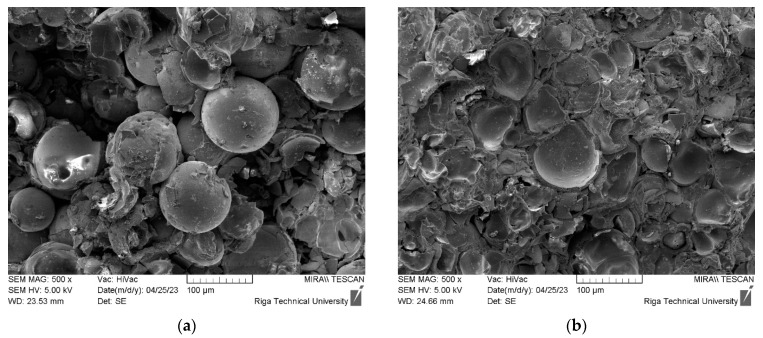
SEM images (magnification X500) of 20 mm samples after compressive strength tests: (**a**) sample sintered at 1050 °C; (**b**) sample sintered at 1180 °C.

**Figure 10:**
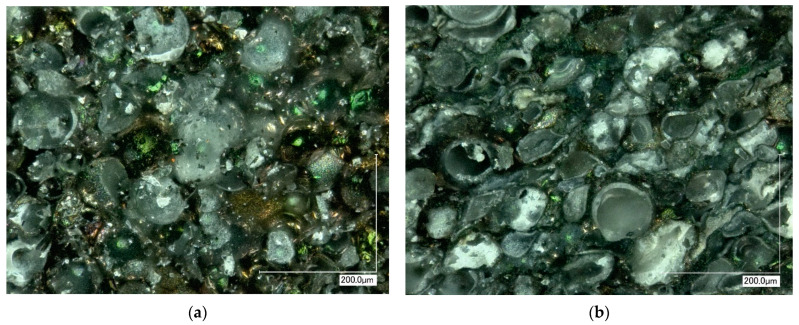
Digital microscope images (magnification X500) of 30 mm samples after compressive strength tests: (**a**) sample sintered at 1050 °C; (**b**) sample sintered at 1200 °C.

The SEM and optical microscope images were captured to examine the morphology of the samples after compression testing (Figure 9 and Figure 10). These images reveal noticeable differences like sample destruction. At lower sintering temperatures, a higher proportion of intact spheres was observed, whereas at higher sintering temperatures, most of the spheres were destroyed. This distinction provided insight into the sample’s destruction mechanism: at lower sintering temperatures, the destruction primarily occurred at the regions where the spheres are bonded together. On the other hand, as the sintering temperature increases, the sintering takes place more effectively, resulting in larger sintered areas between the spheres. Consequently, the destruction of the samples became predominantly associated with the breakdown of individual spheres rather than the bond interfaces between them.

The hereby prepared and characterized material exhibits properties, which make it a promising candidate for EMI shielding applications [23]. 

The concept of introducing and using of direct energy weapon (DEW) which includes EMI and LASER, materials individually for different applications is well known, but combining and loading them to develop a unique material specifically for defense purposes has not, in general, been considered yet. In general, the material should obey several different requirements apart from the EMI and LASER ones (e.g., mechanical, thermal, materials aging, etc.). Additionally, the combination of all these properties (low-density, high-temperature resistant, EMI-depressing) in one material would find use not only for the protection from DEW but also for hypersonic missile parts and as new material for aerospace applications, which is crucial for the use of low-density materials. For the moment it is clear that developing lightweight and at the same time electromagnetic-shielding and high-temperature resistant materials is of high interest. Of course, this material has high demand in civil applications—aerospace. Introducing by Europe, multiple space launchers need new materials that combine their unique properties combinations: low-density, high-temperature resistant, and EMI absorptive. 

## 4. Conclusions

Titanium-coated hollow ceramic spheres were prepared using physical vapor deposition. The powder was then consolidated using an SPS device to obtain bulk cylindrical samples. The as-prepared samples reached density values ranging from 0.9 to 1.8 g·cm^−3^, depending on the sintering temperature (varied from 1050 to 1200 °C). Thermal analyses were performed to evaluate the high-temperature resistance and stability of the samples. Thermogravimetry revealed that the Ti coating was prone to oxidation, which was observed to be run already during the sintering process. The formation of lower Ti oxides (TiO, Ti_2_O_3_) was observed. The aforementioned oxides at high temperatures and in an oxidising atmosphere reacted with the oxygen, and TiO_2_ was formed. Once this process was finished, the oxidation rate was rapidly reduced. The compressive strength of the samples increased with an increasing sintering temperature, due to more intense sintering at high temperatures and reached values of 44–52 MPa (after sintering at 1180 and 1200 °C, respectively). The sample microstructure was compact with a significant amount of open and closed pores. The hereby described material can be a promising candidate for EMI shielding applications. 

## Figures and Tables

**Table 1:** Phase composition of the prepared samples.

Phase ID	Sintering Temperature, °C				
	1050	1100	1150	1180	1200
Mullite	47	50	43	47	51
TiO	4	3	3	3	3
Quartz	3	2	1	2	2
Ti_2_O_3_	1	<1	2	3	4
Rutile	-	-	<1	-	-
Cristobalite	-	-	<1	2	2
Amorphous	45	44	50	43	38

**Table 2:** Results of the EDS analysis of the samples.

Element	Sample ID							
	1050		1100		1150		1180	
	1	2	1	2	1	2	1	2
O	50	47	50	51	50	50	49	52
Al	18	22	23	21	18	25	14	20
Si	28	25	24	23	25	23	28	23
Ti	2	4	1	4	2	1	2	2
K	<1	<1	<1	<1	<1	-	1	<1
Na	<1	<1	-	-	<1	<1	2	<1
Ca	<1	-	-	-	2	-	<1	2
Mg	-	-	-	-	-	-	<1	-
Fe	-	<1	<1	-	<1	-	2	-

## Data Availability

Data are contained within the article.
